# Evaluation of five column‐based isolation kits and their ability to extract miRNA from human milk

**DOI:** 10.1111/jcmm.16726

**Published:** 2021-06-27

**Authors:** Emelie Ahlberg, Maria C. Jenmalm, Lina Tingö

**Affiliations:** ^1^ Division of Inflammation and Infection Department of Biomedical and Clinical Sciences Linköping University Linköping Sweden; ^2^ Örebro University Food and Health Programme, School of Medical Sciences Örebro University Örebro Sweden

**Keywords:** breast milk, microRNA, milk fraction, qPCR, RNA extraction, skim milk, small non‐coding RNA

## Abstract

MicroRNA can be found in various body fluids, including breast milk. MicroRNA may be transferred from mother to infant via breast milk and potentially regulate the development of the infant's immune system on a post‐transcriptional level. This study aimed to determine the microRNA extraction efficiency of five RNA extraction kits from human skim milk samples. Their efficiency was determined by comparing microRNA concentrations, total RNA yield and purity. Furthermore, hsa‐miR‐148a‐3p expression and the recovery of an exogenous control, cel‐miR‐39‐3p, were quantified using qPCR. Each kit extracted different amounts of microRNA and total RNA, with one kit tending to isolate the highest amount of both RNA species. Based on these results, the extraction kit ReliaPrep™ miRNA Cell and Tissue Miniprep System from Promega was found to be the most appropriate kit for microRNA extraction from human skim milk. Moreover, further research is needed to establish a standardized protocol for microRNA extraction from breast milk.

## 1 INTRODUCTION

Several factors in breast milk (BM), *for example* immunoglobulins, have immunomodulatory effects.^1^ Interestingly, BM also harbours a vast array of small RNA species that can act as an alternate, and less‐explored route, to facilitate immune programming in infants.^2^, ^3^ miRNAs are very short RNA molecules (18‐22 nucleotides long) that could potentially influence immune maturation through direct effects, *for example* by inhibiting expression of key transcription factors for T cell polarization.^2^, ^3^ The miRNA that presents in BM is believed to be produced by the mammary epithelial cells, subsequently encapsulated in exosomes and released into the fluid.^4^ Milk exosomes, containing the miRNAs, may then be absorbed by the offspring with maintained biological function and be distributed to a range of organs via the circulation.^5^

Efficient isolation of miRNAs from biofluids is essential for downstream studies of their functional capacities. However, as standardized protocols for BM miRNA extraction are lacking, it is critical to establish appropriate methodology as has been done for other biofluids like plasma^6^, ^7^ and serum.^8^, ^9^ A paper from 2015 has compared different RNA extraction kits, including phenol/chloroform, combined phenol column and column filter‐based kits in BM; the findings suggest that phenol‐free kits should be the primary choice for isolating miRNA.^10^ Therefore, we aimed to investigate miRNA extraction efficiency in human BM in a set of chloroform‐ and phenol‐free RNA extraction kits that are currently available on the international market.

## 2 MATERIALS AND METHODS

### 2.1 Sample collection and milk fractioning

**FIGURE 1:**
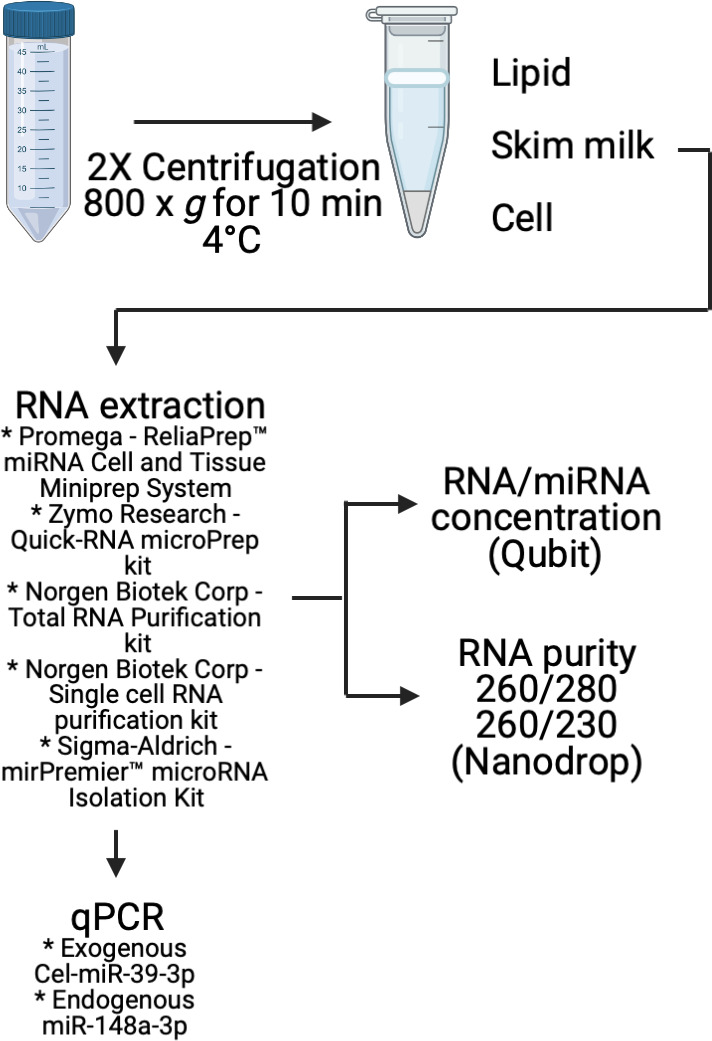
Overview of the experimental design

BM was collected at the first morning feeding by ten healthy women 4 months post‐partum and stored at −20°C. After transportation to the university, all samples were kept at −70°C until analysis. The milk was thawed and fractionated by centrifugation, 800 × g, 10 minutes, 4°C; skim milk was further purified by repeating the centrifugation in new tubes, see Figure 1. The study was approved by the Regional Ethics Committee in Linköping, Dnr 2011/45‐31.

### 2.2 Kits and RNA extraction

This study investigates the performance of five phenol/chloroform‐free RNA isolation kits that, at the time of the study, were commercially available on the international market.

Although the manufacturers' protocols were generally followed, some modifications were established, as shown in Appendix 1. Two µL (250 pM) of cel‐miR‐39‐3p from *Caenorhabditis elegans* (Cat# 4464066, Invitrogen) was added before extraction, and the elution step was repeated to extract as much miRNA as possible from the matrix.

### 2.3 Quantification and purity

RNA quantification was performed using Qubit microRNA Assay kit and Qubit RNA HS Assay kit together with the Qubit 3.0 Fluorometer (Invitrogen), following guidelines; Nanodrop ND‐1000 spectrophotometer (Thermo Fisher Scientific) was used for purity assessment.

### 2.4 miRNA recovery

The exogenous miRNA cel‐miR‐39‐3p and the endogenous miRNA hsa‐miR‐148a‐3p were quantified by qPCR. cDNA template was synthesized using TaqMan Advanced miRNA cDNA Synthesis Kit (Applied Biosystems), following the manufacturers' guidelines. The qPCR product was preformed using TaqMan Fast Advanced Master Mix (Applied Biosystems), TaqMan Advanced miRNA Assay primers cel‐miR‐39‐3p (Applied Biosystems, Assay ID 478293_mir) and hsa‐mir‐148a‐3p (Applied Biosystems, Assay ID 477814_mir). The reactions were processed in a 7500 Fast Real‐Time PCR instrument (Applied Biosystems) using the following settings: 95°C for 20 seconds, followed by 40 cycles of 95°C for 3 seconds and 60°C for 30 seconds. All reactions, including the non‐template controls, were run in duplicates. Ct values were determined using fixed‐threshold and analysed using the Thermo Fisher Connect Software (Thermo Fisher Scientific) available online.

### 2.5 Statistics

Statistical analysis and data visualization were performed using GraphPad prism version 7.04 (GraphPad software, Inc). The performance of the extraction kits was compared in terms of (a) their miRNA and total RNA recovery, (b) their hsa‐miR‐148a‐3p yield and (c) their ability to recover the exogenous cel‐miR‐39‐3p. Wilcoxon matched‐pairs signed rank test was used to evaluate statistical significance between the extraction kits. Two outliers were removed from the RNA purity data using the ROUT method^11^ before further comparisons using Wilcoxon matched‐pairs signed rank test. *P* values of <.05 were considered significant.

## 3 RESULTS

### 3.1 RNA concentration

Refer to Table 1 for median and range.

**TABLE 1:** Comparison of total RNA and miRNA concentrations between five RNA extraction kits

Extraction kit	Total RNA (ng/µL)	microRNA (ng/µL)	RNA purity (OD 260/280)	RNA purity (OD 260/230)
Promega	28.30, 7.68‐52.65	12.75, 3.35‐56.65	1.94, 1.68‐2.03	0.52, 0.17‐0.81
Zymo	20.30, 8.47‐28.45	10.60, 3.72‐34.70	1.35, 1.30‐1.56	0.32, 0.25‐0.39
Norgen RNA	23.10, 10.70‐34.05	8.40, 3.99‐45.35	1.48, 1.32‐1.71	0.24, 0.11‐0.35
Norgen cell	6.20, 0.02‐6.89	1.92, 1.02‐22.92	1.53, 1.31‐1.75	0.14, 0.05‐0.28
Sigma‐Aldrich	14.16, 3.97‐38.25	3.99, 0.85‐37.95	2.20, 2.08‐2.29^a^	1.48, 0.79‐2.07

Note

Promega – ReliaPrep™ miRNA Cell and Tissue Miniprep System (n = 10); Zymo Research – Quick‐RNA microPrep kit (n = 10); Norgen Biotek Corp ‐ Total‐RNA Purification kit (n = 8) and Single Cell RNA purification kit (n = 8); Sigma‐Aldrich – mirPremier™ microRNA Isolation Kit (n = 10). RNA was extracted from human skim milk and measured using Qubit 3.0. RNA purity was estimated by measuring the absorbance at 260 nm, 280 nm and 230 nm, using Nanodrop ND‐1000. A 260/280 ratio of ~2.0 is generally accepted as ‘pure’ for RNA and expected 260/230 ratios are commonly in the range of 2.0‐2.2. All values are presented as median and interquartile range (25th‐75th percentile).

^a^ Two outliers have been removed, after using the ROUT method.

The total RNA concentration after extraction with Norgen Single Cell (NSC) kit was lower compared with Promega, Zymo and Norgen RNA (NR) (*P* < .05), and the Promega kit yielded higher concentrations compared with Zymo (*P* < .05).

The NSC kit had lower miRNA yield compared with Promega, Zymo and NR (*P* < .05), the Promega kit yielded higher concentrations compared with Zymo and Sigma‐Aldrich (*P* < .05), and NR yielded higher concentrations than that of the Sigma‐Aldrich kit (*P* < .05).

### 3.2 Purity

Only the Promega and Sigma‐Aldrich kits yielded reasonable 260/280 ratios, Table 1. The Promega kit had a higher 260/280 ratio than Zymo and both Norgen kits (*P* < .01), and the Sigma‐Aldrich kit had a higher ratio than Zymo (*P* < .05). All the included kits produced 260/230 ratios <1 except the Sigma‐Aldrich kit, which was higher than the other kits (*P* < .01). Also, a higher 260/230 ratio was observed in the Promega kit compared with the NSC (*P* < .05).

### 3.3 Extraction efficiency

**FIGURE 2:**
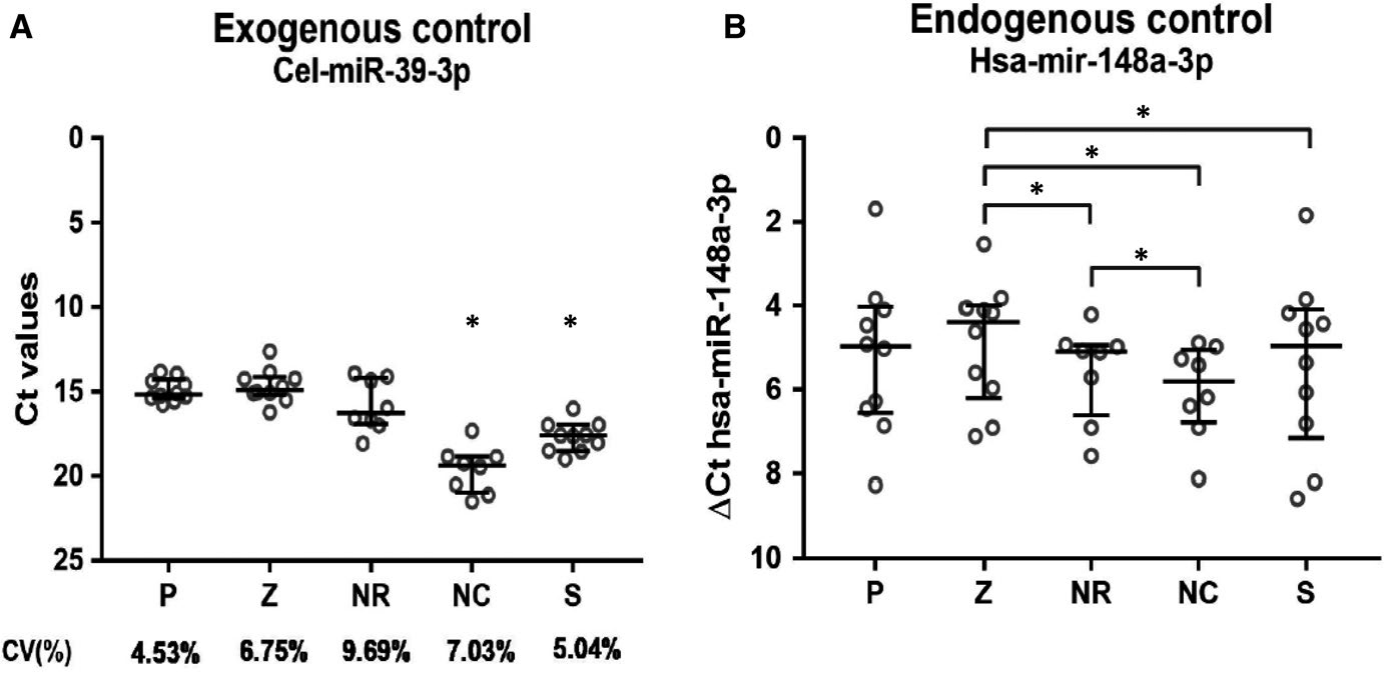
miRNA recovery. P = Promega – ReliaPrep™ miRNA Cell and Tissue Miniprep System (n = 10); Z = Zymo Research – Quick‐RNA microPrep kit (n = 10); NR = Norgen Biotek Corp – total‐RNA Purification kit (n = 8); NC = Norgen Biotek Corp – Single Cell RNA purification kit (n = 8); S = Sigma‐Aldrich – mirPremier™ microRNA Isolation Kit (n = 10). A low Ct value indicate higher miRNA recovery and a low coefficient of variance (CV%) indicate low extraction variation between samples. Error bars indicate median and interquartile range (25th‐75th percentile). Wilcoxon matched pairs signed rank test was used to evaluate statistical significance between the extraction kits. *P value <.05. A, shows the cel‐miR‐39‐3p recovery: Kit P, Z and recovered similar levels of the exogenous control (median Ct of 15.16, 14.89 and 16.28, respectively) and significantly more compared to NC and S (median Ct of 19.37 and 17.62, respectively). No significant difference between kit NC and S was observed. B, shows the hsa‐miR‐148a‐3p recovery. Calculation of ΔCt hsa‐miR‐148a‐3p was done using the following equation; ΔCt = Ct (hsa‐miR‐148a‐3p) – Ct (Cel‐miR‐39‐3p), for each sample. High ΔCt value indicates low recovery of the endogenous control

The Promega, Zymo and NR kits recovered similar amounts of cel‐miR‐39‐3p (Figure 2), showing better recovery than the NSC and Sigma‐Aldrich kits (*P* < .05). The Zymo kit recovered the most hsa‐miR‐148a‐3p, significantly more than the NR, the NSC and the Sigma‐Aldrich kits (*P* < .05). No difference was found between Zymo and Promega, or the Promega and Sigma‐Aldrich kits; NSC had the lowest recovery of all five kits.

## 4 DISCUSSION

miRNA is present in various biofluids and in high quantity in BM, where it has a potential role in regulating gene expression in the breastfed infant.^3^ However, no standardized protocols for isolation and quantification of breast milk miRNA are currently available, which will be necessary to facilitate further studies of their biological function. The miRNA extraction kits currently available on the market are in general designed for biofluids like plasma and serum or for cells. This calls for evaluation of extraction kits compatibility with BM specifically, to facilitate further studies investigating this particular biofluid. Although one previous study by Alsaweed et al^12^ compared the performance of eight extraction kits in BM, the main focus of that paper was to compare miRNA retrieval between the different milk fractions (lipids, cells and skim milk). Furthermore, the kits were also compared in terms of total RNA yield and purity, and lastly the ratio of miRNA and small RNA. In addition, some studies have compared the isolation capacity of commercially available miRNA extraction kits in other bodily fluids, such as plasma,^7^ serum ^8^ and blood cells.^9^, ^13^ These previous studies have, however, to a large extent included kits requiring phenols and/or chloroform. If inhaled, these volatile substances will have severe negative effects on human health and they are hence troublesome to work with; particular safety measures will be needed. In addition, they may leave traces in the samples that can pose a problem in downstream analyses.^14^ In this study, we evaluated the performance of five chloroform‐ and phenol‐free column‐based RNA extraction kits to investigate their compatibility with BM. Total RNA and miRNA concentration varied between the individuals and kits, with the kits from Promega and Zymo extracting the highest levels of total RNA and miRNA, whereas the poorest performance was observed for the Norgen Single cell kit. The kits from Promega and Zymo also recovered most of the exogenous and endogenous control. Concerning RNA purity, all kits produced rather poor 260/280 and 260/230 ratios; the kit from Sigma‐Aldrich produced the purest samples. The Sigma‐Aldrich kit was also evaluated in the study by Alsaweed et al^10^ and produced a mean 260/280 ratio of 1.4; this is lower than what we found in our study. However, as mentioned previously, samples with low miRNA and total RNA concentrations will have a lower absorbance. Thus, spectrophotometric methods might not be sensitive enough to properly evaluate this.

Even though none of the kits we tested here were specifically designed to be compatible with BM, the majority of the kits performed reasonably well. The varying performance of the evaluated kits may be due to a number of factors that might influence the kits' ability to extract miRNA. Firstly, the input volume may affect the extraction capacity as some kits have a low RNA binding capacity, *for example* the Norgen Single Cell kit. In such cases, the excess miRNA may have been washed away. Secondly, the effectiveness of the column matrix binding capacity may vary. Although the matrices are most commonly constituted of silica, both the Norgen kits in this study used silicon carbide. The Norgen Total RNA kit had quite high miRNA yield, which might be attributed to the binding capacity of the matrix.^15^ In contrast, the Norgen Single Cell kit performed poorly on most parameters investigated in this study and was not particularly well suited for extracting miRNA from BM. Also, the matrix of the Promega kit was silica‐based, and this kit performed in general better than Norgen Total RNA on all parameters, thus providing no basis to regard the silicon carbide matrix as superior to silica when extracting miRNA from BM.

Moreover, variations in the performance of the kits might have been due to differences in the lysis step. The effectiveness of the lysis buffer is an important step to release RNA and miRNA that is not already in solution and to stabilize RNA molecules, including inhibition of RNase activity. In BM, this would, for example, include RNAs that are trapped in extracellular vesicles, *for example* exosomes. The kits varied in their suggested volumes of samples and solutions; in addition, the reagents in the kits had somewhat varying composition. Together, these factors contribute to small but important differences, *for example* in lysis and binding abilities, and thus limit comparability between the kits and between different studies. In the optimization process proceeding the laboratory work for this study, we made some minor changes to the protocols in order to make them work more efficiently with our samples. Only the Norgen kits had a protocol for biofluids and did not need any major modifications; we followed the protocol described for blood. The other kits, however, were primarily designed for RNA extraction from cells. Hence, we modified the lysis step slightly by adjusting the amount of ethanol added and disregarded the steps for purification of sample from cellular debris. We, however, followed the proportion stated for lysis and ethanol for each kit. It is possible that, although we increased performance with these set of changes, the increased exposure of the samples to ethanol may have contributed to the lower than expected RNA quality in our isolates. However, in our case when we have a low miRNA concentration and likely salt contamination from the lysis buffer, spectrophotometry is potentially a less suitable method to estimate RNA quality.

The Nanodrop measurements of RNA purity are evaluated by the ratio of absorbance at the 260/230 nm and the 260/280 nm spectrum. This is because nucleotides, *that is* RNA and DNA, will absorb at 260 nm and common contaminants will appear at the other wavelengths.^14^ The 260/230 ratio for ‘pure’ nucleic acid is commonly in the range of 2.0‐2.2. If the ratio is lower than expected, this may indicate the presence of contaminants absorbing at 230 nm. Commonly used additives, or residues, with absorbance near the 230 nm spectrum are carbohydrates, EDTA and phenol. The RNA isolation kits in this study were free from the latter two substances. BM is, however, a rather rich carbohydrate source, and the guanidinium salt, which is the main component in the lysis buffers, has its absorbance at 230 nm and could potentially be the primary cause for the low 260/230 ratios in this study. In many RNA extraction kits, however, the guanidine isothiocyanate is more common and this will absorb at ~280 nm. Furthermore, a 260/280 ratio of ~2.0 is generally accepted as ‘pure’ for RNA. Most of the isolation kits in this study, except for the Sigma‐Aldrich kit, came below this ratio. If the ratio is appreciably lower, as in this case, it indicates the presence of contaminants that absorb near 280 nm. Such contaminants could, for example, be phenol or proteins, which however are not likely the case in this study as the kits where phenol‐free and involve a number of filtering steps to remove larger particles. Small changes in the pH of the solution may also cause the 260/280 to vary.^16^ Acidic solutions can under‐represent the 260/280 ratio, although a basic solution may over‐represent it. Although we did not check our samples for pH, BM 4 months post‐partum is in the pH neutral range.^17^ Moreover, the five nucleotides that comprise RNA exhibit widely varying 260/280 ratios.^18^ The following represent the 260/280 ratios estimated for each nucleotide if measured independently: Guanine: 1.15; Adenine: 4.50; Cytosine: 1.51; Uracil: 4.00; Thymine: 1.47. The resultant 260/280 ratio for the nucleic acid being studied will be approximately equal to the weighted average of the 260/280 ratios for the four nucleotides present. There are, however, indications from previous studies that miRNAs of exosomal origin are particularly rich in Guanine bases.^19^ As Guanine is one of the five bases producing the lowest 260/280, this may be of significance to our samples, as BM is rich in exosomes. It is, hence, important to note that the generally accepted purity ratios of between 1.8 and 2.0 are ‘rules of thumb’ as the actual ratio will depend on the composition of the nucleic acid. However, as we evaluated the purity ratios in the same samples, all five kits would likely have produced equally low ratios if the low values were due to the sample characteristics and not kit performance. Lastly, one should point out that spectrophotometry methods such as Nanodrop are not particularly sensitive in the low concentration spectrum and are more suited for higher concentrated samples with longer RNA species. In our case, low RNA concentration and guanidinium salt contamination are likely the cause of the low RNA purity in our samples.

One strength with this study is that this is the first study, to the best of our knowledge, that uses Qubit for miRNA quantification and not exclusively the Bioanalyzer or Nanodrop in the BM field. We chose to include this method because of the previously reported high selectively and reproducibility of Qubit, as compared to Bioanalyzer and Nanodrop.^20^ Indeed, we found the Qubit to be sensitive and reliable with a high reproducibility in its measurements, both between samples and between readings of the same samples at multiple occasions (data not shown). Surprisingly, for some samples the concentration of miRNA was higher compared to total RNA. This might be explained by the high selectively of Qubit for measuring only miRNA (sequences below 40 bases in length) when preparing the samples with the miRNA assay, and the selectivity of the RNA HS assay for bigger RNA molecules (>20 bases). Hence, we might have had an underrepresentation of the miRNA fraction >20 bases in the total RNA measurements. This possible underrepresentation of short RNA sequences might be important to consider also for other measurements of total RNA, for example when estimating the needed amount of RNA to be deployed for downstream analyses. Rather than basing the input estimations for such analyses on total RNA or small RNA (~70‐200 bases), which is more the actual readout of the Nanodrop and the Bioanalyzer, a Qubit measurement of miRNA would be more representative. A further limitation with Nanodrop, as compared to Qubit, is that it quantifies the RNA based on absorbance at 260 nm, including all nucleotides present in the sample meaning that the Nanodrop will also take into account fragmented RNA. Moreover, even though the Bioanalyzer may be more selective for small RNA as compared to the Nanodrop, Garcia‐Elias et al^20^ observed low reproducibility, also limiting accurate quantification. Furthermore, Qubit tolerates contaminants, such as salts, better than the Nanodrop, which may be of significance when the samples are likely to contain chemical residues as discussed above.

Lastly, one additional strength with this study is that an exogenous control, cel‐miR‐39‐3p, was added in a standardized amount to all samples prior to the RNA extraction. This control allowed for normalization of technical variability between the samples and was used to assess miRNA recovery. A low coefficient of variance (CV) between the samples within a kit would indicate consistency in the amount of miRNA recovered; in this study, Promega had the lowest CV (4.53%) of the included kits.

## 5 CONCLUSION

In conclusion, our results suggest that at least two of the extraction kits, the ones from Promega and Zymo, were reasonably efficient in terms of recovery and consistency. Acceptable 260/280 ratios were also observed for the Promega kit, although this kind of spectrophotometry method is not so well suited to evaluate purity in miRNA samples. As the Promega kit was also one of the best performers on the other investigated parameters (*ie* total RNA extraction efficiency, extraction of hsa‐miR‐148a‐3p and recovery of cel‐miR‐39‐3p), as of today, this would be our primary choice for miRNA extraction from BM. For future validation studies, it is important to keep in mind that the input volume must be standardized, as must the elution volume, to produce comparable results between kits. A spike‐in of an exogenous miRNA is also needed to control for technical variability and for evaluating miRNA recovery. The exogenous control should also be used to normalize levels of endogenous miRNAs and is particularly important in the absence of reliable reference miRNAs comparable to the ‘housekeeping genes’ utilized in mRNA qPCR. Furthermore, we would recommend the Qubit miRNA assay as a reliable, sensitive and reproducible method to estimate miRNA content in RNA isolates.

## CONFLICT OF INTEREST

The authors confirm no conflicts of interest.

## AUTHOR CONTRIBUTIONS

**Emelie Ahlberg:** Conceptualization (equal); Data curation (lead); Formal analysis (lead); Investigation (equal); Methodology (equal); Visualization (equal); Writing‐original draft (equal); Writing‐review & editing (equal). **Maria C Jenmalm:** Project administration (supporting); Supervision (supporting); Writing‐review & editing (supporting). **Lina Tingö:** Conceptualization (equal); Data curation (supporting); Formal analysis (supporting); Funding acquisition (lead); Investigation (equal); Methodology (equal); Project administration (lead); Supervision (lead); Writing‐original draft (equal); Writing‐review & editing (equal).

## Data Availability

The data that support the findings of this study are available from the corresponding author upon reasonable request.

## APPENDIX 1

RNA extraction protocols with modifications

**Sigma mirPremier microRNA Isolation Kit**

The protocol provided with the kit was modified as follows: 126 µL Lysis buffer was mixed with 64 µL binding solution and 10 µL 100% ethanol. 100 µL sample was lysed with 2 volumes lysis buffer, followed by vortexing for 15 s and incubation for 5 min in room temperature. 1.1 volume of 100% ethanol was added to the lysate, followed by vortexing for 15 s. The lysate was transferred to a Binding column, followed by centrifugation at 16 000 × *g* for 30 sec. The flow‐through was discarded, and 700 µL of 100% ethanol was added to the column, followed by centrifugation at 16 000 × *g* for 30 s. The flow‐through was discarded and 500 µL binding solution was added, followed by centrifugation at 16 000 × *g* for 30 s. The column was transferred to a new collection tube and was washed twice with 500 µL Washing solution, followed by centrifugation at 16 000 × *g* for 30 s. The flow‐through was discarded and the column was dry‐centrifuged at 16 000 × *g* for 1 min. The column was transferred to a new tube and 20 µL RNase‐free water was added, followed by 1‐min incubation in room temperature and later centrifuge at 16 000 × *g* for 1 min. To maximize the miRNA recovery, a second elution was performed with the eluted RNase‐free water.

**Zymo Quick‐RNA MicroPrep Kit**

The protocol provided with the kit was modified as follows: 100 µL sample was lysed with 2 volumes of lysis buffer, followed by vortexing for 15 s. Two volumes of 99.5% ethanol was added to the lysate followed by vortexing for 10 s, before it was transferred to a Zymo‐Spin™ IIICG Column and centrifuged at 12 000 × *g* for 30 s. The flow‐through was discarded and 400 µL of RNA Prep Buffer was added to the column before centrifugation at 12 000 × *g* for 30 s. The flow‐through was discarded, and 700 µL RNA wash buffer was added to the column before centrifugation at 12 000 × *g* for 30 s. The flow‐through was discarded, and the washing step was repeated by adding 400 µL RNA wash buffer, before centrifugation at 12 000 × *g* for 2 min, followed by dry centrifugation of the column at 12 000 × *g* for 1 min. The column was transferred to a new tube and 20 µL RNase‐free water was added to elute the miRNA, followed by centrifugation at 16 000 × *g* for 30 s. To maximize the miRNA recovery a second elution was performed with the eluted RNase‐free water.

**Promega** **ReliaPrep miRNA Cell and Tissue Miniprep System**

The protocol provided with the kit was modified as follows: 100 µL sample was lysed using 3 volumes lysis/1‐Thioglycerol buffer and 600 µL 95% ethanol was added to the lysate, followed by vortexing for 10 s. The lysate was transferred to a ReliaPrep™ Minicolumn, followed by centrifugation at 12 000 × *g* for 30 s. The flow‐through was discarded and 500 µL wash buffer was added to the column, followed by centrifugation at 12 000 × *g* for 30 s. The flow‐through was discarded and 500 µL wash buffer was added to the column, followed by centrifugation at 12 000 × *g* for 2 min. The column was transferred to a new tube and 20 µL RNase‐free water was added, followed by centrifugation at 12 000 × *g* for 1 min. To maximize the miRNA recovery, a second elution was performed with the eluted RNase‐free water.

**Norgen Total RNA Purification kit/Single Cell and Tissue Miniprep System**

The protocol provided with the Norgen kits were modified as followed: 100 µL sample was lysed using 3.5 volumes of Buffer RL, followed by vortexing for 15 s. 200 µL 99.9% ethanol was added to the lysate, followed by vortexing for 10 s. The lysate was transferred to the column and centrifuged at 3500 × *g* for 1 min. The flow‐through was discarded and 400 µL wash solution A was added to the column, followed by centrifugation at 14 000 × *g* for 1 min. The flow‐through was discarded, and the same washing step was preformed twice. After the third wash‐step, the flow‐through was discarded, followed by 2 min dry‐centrifugation of the column at 14 000 × *g*. The column was transferred to a new tube and 50 µL (Total RNA) or 20 µL (Single Cell) Elution solution A was added to elute the miRNA followed by centrifugation at 200 × *g* for 2 min and 1 min at 14 000 × *g*. To maximize the miRNA recovery, a second elution was performed with the eluted sample.

*Validation of RNA purity extracted from cell lysate*

Methods

RNA was extracted from human mammary epithelial cells (Gibco, Thermo Fisher Scientific, MS, USA) using Zymo Quick‐RNA MicroPrep Kit (n = 3) and Promega ReliaPrep miRNA Cell and Tissue Miniprep System (n = 2) according to the manufactory's instructions. In brief, cells were cultured in HuMEC ready medium (Gibco), supplemented with Bovine Pituitary Extract (Gibco), HuMEC Supplement (Gibco) and 30 µg/mL of Penicillin‐Streptomycin (Gibco) until 80%‐90% confluence. Medium was removed, and cells were washed with sterile PBS and prewarmed TrypLE (Invitrogen, Thermo Fisher Scientific, MS, USA), without phenol red was added and incubated for 15 min in 37℃. Cell count and viability were determined, using Countess II Automated Cell Counter (Invitrogen) with 0.4% Trypan Blue (Gibco). 1 × 10^6^ cells were lysed with corresponding lysis solution, and RNA was eluted with 100 µL and 30 µL RNase‐free water, respectively. RNA concentration and purity were estimated using Nanodrop ND‐1000 spectrophotometer, by evaluating the A260/280 ratios.

Results

RNA concentration ranged between 166.8‐274.6 ng/µL (Zymo) and 283‐298 ng/µL (Promega). RNA purity based on the A260/280 ratios ranged between 2.12‐2.14 (Zymo) and 1.85‐2.15 (Promega).

*Disclosure*

The two kits from Norgen Biotek Corp were provided to our laboratory as free samples.
